# Synthesis and application of quercetin hybrids as potential anticancer agents in the treatment of ovarian cancer

**DOI:** 10.1038/s41598-026-50954-9

**Published:** 2026-04-28

**Authors:** Dominika Wendlocha, Monika Kadela-Tomanek, Paweł Ramos, Robert Kubina, Kamil Krzykawski, Patryk Chudy, Aleksandra Mielczarek-Palacz

**Affiliations:** 1 Department of Immunology and Serology, Faculty of Pharmaceutical Sciences in Sosnowiec, Medical University of Silesia in Katowice, 41-200 Sosnowiec, Poland; 2 Department of Organic Chemistry, Faculty of Pharmaceutical Sciences in Sosnowiec, Medical University of Silesia in Katowice, 4 Jagiellońska Str., 41-200 Sosnowiec, Poland; 3 Department of Community Pharmacy, Faculty of Pharmaceutical Sciences in Sosnowiec, Medical University of Silesia in Katowice, Jedności 8 B, 41-200 Sosnowiec, Poland; 4 Department of Pathology, Faculty of Pharmaceutical Sciences in Sosnowiec, Medical University of Silesia in Katowice, Ostrogórska 30, 41-200 Sosnowiec, Poland; 5 Silesia LabMed: Research and Implementation Centre , Medical University of Silesia in Katowice, 18 Medyków Str., 40-752 Katowice, Poland; 6 Doctoral School, Medical University of Silesia in Katowice, 15 Poniatowskiego Str., 40-055 Katowice, Poland

**Keywords:** Ovarian cancer, Quercetin, Flavonoids, Biochemistry, Cancer, Chemistry, Drug discovery

## Abstract

Cancers, including ovarian cancer, are a growing public health problem due to their high incidence and mortality rates. Ovarian cancer is a particularly challenging neoplasm to diagnose in its early stages due to the absence of specific symptoms and the paucity of effective diagnostic methods. The standard treatment regime for this condition includes surgical intervention, the administration of chemotherapy, and radiation therapy. Flavonoids, a prevalent class of phytonutrients, have been demonstrated to possess a plethora of biological properties, including antioxidant, anti-inflammatory, and anticancer properties. Quercetin, a widely recognised flavonol, has garnered significant attention due to its limited bioavailability, which has prompted the exploration of novel, more active derivatives. Research has demonstrated that structural modifications, incorporating the incorporation of groups comprising single, double or triple bonds, can substantially augment anticancer activity by modulating apoptosis, inhibiting angiogenesis and constraining cancer cell migration. In the course of the study, a series of new quercetin derivatives were synthesised, and their effects on ovarian cancer cells and normal cells were evaluated. Two compounds were selected from among those obtained, which showed particularly strong activity against the OVCAR-3 and SW626 cell lines. Furthermore, thermogravimetric analysis (TGA) was employed to ascertain the thermal stability and physicochemical properties of the compounds under investigation.

**Supplementary Information:**

The online version contains supplementary material available at 10.1038/s41598-026-50954-9.

## Introduction

Cancer diseases and cancer syndromes constitute a significant public health problem worldwide. Epidemiological reports indicate a steady increase in the incidence of cancers, including ovarian cancer^1^. Ovarian cancer (OC) is considered the most lethal gynecological malignancy, characterized by numerous genetic mutations that trigger uncontrolled cell growth and replication^2^. The symptoms of ovarian cancer are nonspecific, and its early detection is difficult due to the lack of effective diagnostic tools. The most common treatment methods for ovarian cancer include surgery, chemotherapy, and radiotherapy, used alone or in combination^3^.

Flavonoids are a group of micronutrients widely distributed in plant-derived products. Numerous studies have demonstrated that these compounds possess a broad spectrum of biological activities, including anticancer, antioxidant, and anti-inflammatory effects^4^. The chemical structure of flavones consists of two benzene rings (designated as A and B) linked by a third heterocyclic ring containing an oxygen atom (ring C). A carbonyl group is located at the C4 position, contributing to an increase in the molecule’s polarity. One of the most widespread subclasses of flavones is the flavonols, which are characterized by the presence of a hydroxyl group at position 3 and a double bond between the C2 and C3 atoms^5^.

Quercetin (QUE) (3,3′,4′,5,7-pentahydroxyflavone) is the principal representative of the flavonol subclass. It is widely distributed in fruits and vegetables, occurring in significant amounts in broccoli, lettuce, and onions. In plants, quercetin is predominantly present in the form of glycosides, which are hydrolyzed in the intestine by the enzyme β-glucosidase through the cleavage of glycosidic bonds, followed by absorption by enterocytes. Subsequently, quercetin undergoes metabolism to form conjugates with increased water solubility.

Quercetin (QUE), like other flavonols, exhibits numerous biological properties, including antioxidant, anti-inflammatory, and anticancer activities. Its biological activity has been widely described in the literature, although its effectiveness may depend on various factors, including bioavailability[6-7- ^8–10^. Unfortunately, quercetin is characterized by low bioavailability, which consequently affects its biological activity and may explain the discrepancies observed between in vitro and in vivo studies^11^.

This has led to the synthesis of novel quercetin derivatives, which may exhibit enhanced biological activity and improved physicochemical properties, including the ability to form complexes with metal ions^11,12^.

**Fig. 1:**
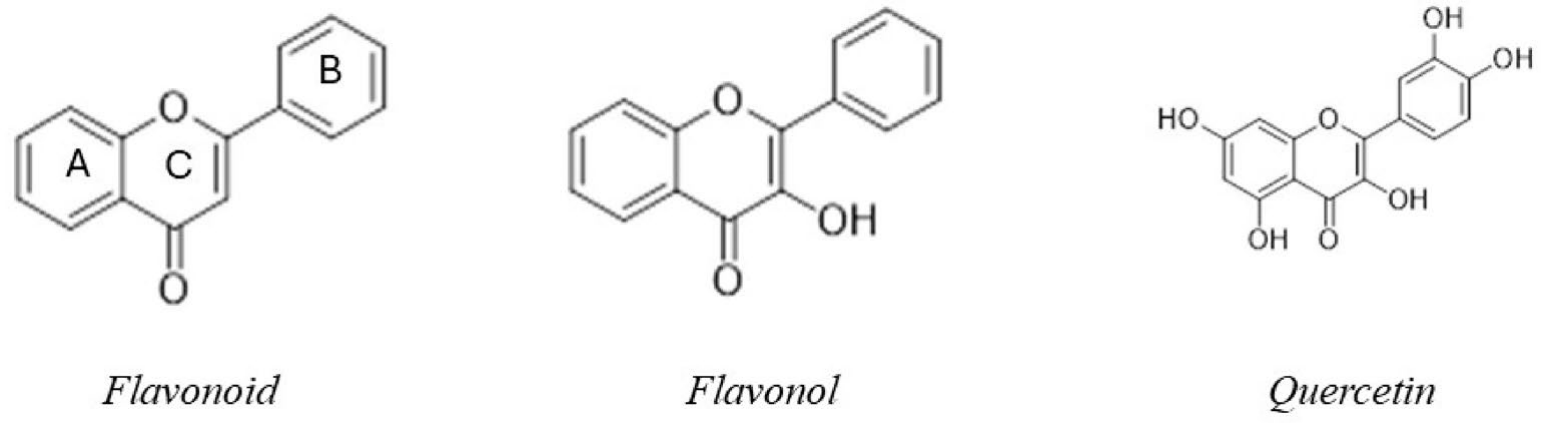
The chemical structure of flavonoid, flavanol and quercetin.

In a study by Zhang et al.^13^, quercetin derivatives with quinoline scaffolds were synthesized, demonstrating increased anticancer activity against liver cancer cells. Similar observations have been reported for water-soluble quercetin metabolites, such as quercetin 3′-sulfate (Q3’S) and quercetin-3-glucuronide (Q3G), which showed dose-dependent inhibition of breast cancer cell proliferation^14^. Furthermore, various quercetin derivatives have been reported to influence cancer cell behavior, including proliferation, migration, and invasiveness, depending on their chemical structure and functional groups^15–17^.( Figure 1) 

Studies on modified quercetin derivatives have also shown that the introduction of different substituents can significantly affect their biological activity. In particular, the position and type of substituent may influence the overall effectiveness of these compounds, highlighting the importance of structural modifications in the development of new quercetin-based agents^16,17^. One approach to modifying the structure of natural compounds is the introduction of additional substituents. The chemical properties and biological activity of the newly synthesized compounds depend on the type of bonds present in the substituent (single, double, or triple). The incorporation of one or more π-bonds enhances the electron-withdrawing inductive effect of the carbon atom and increases molecular reactivity, which can influence metabolism or alter the profile of adverse effects^18^. Furthermore, a substituent containing a double or triple bond reduces molecular flexibility, which can affect the interaction of the compound with its biological target and, consequently, its activity^19^. In literature described a few O-alkyl and O-acyl derivatives of quercetin which characterized anticancer and antiviral activity. The aim of the present study is the synthesis of quercetin derivatives containing substituents with single, double, or triple bonds. Literature data suggest that quercetin derivatives are highly promising chemopreventive agents due to their enhanced bioavailability and low toxicity. The anticancer activity of the newly synthesized compounds was evaluated in vitro using ovarian cancer cells as well as normal cells.

Thermogravimetric analysis (TGA) is one of the key analytical techniques used to characterise the thermal behaviour and stability of newly synthesised compounds^20,21^. In the development of biologically active and potentially therapeutic substances, thermal analysis provides essential information about the degradation profile, phase transitions, and purity of materials^22,23^. The determination of thermal stability is particularly important for pharmaceutical candidates, as it directly influences their processing, formulation, storage, and shelf life^21,24,25^.

The evaluation of mass loss as a function of temperature allows for the identification of decomposition stages and the assessment of the physicochemical robustness of new compounds under thermal stress^26,27^. When combined with complementary methods such as differential thermal analysis (DTA) or differential scanning calorimetry (DSC), TGA enables a more comprehensive understanding of the energetics of thermal events, including melting, crystallization, or decomposition processes^21–26^.

In the context of drug design and materials chemistry, thermogravimetric studies serve not only as a diagnostic tool but also as a predictive approach for assessing compound stability and compatibility with excipients^20,21,28^. Therefore, the application of thermal methods is a crucial step in the preformulation and characterization of new substances with potential therapeutic activity.

## Materials and method

### Synthesis of derivatives 2–5

Electrothermal IA 9300 melting point apparatus was used to determine the melting points. Bruker Impact II instrument (Bruker, Billerica, MA, USA) was used to record the high-resolution mass spectral analysis (HR-MS). The spectra were visualised using the Bruker Compass DataAnalysis 4.3 software. The theoretical value of molecular weight was determined using the online available Exact Mass Calculator^1^. Bruker Avance 600 spectrometer (Bruker, Billerica, MA, USA) was used to measure the nuclear magnetic resonance (NMR) spectra. Chemical shifts (δ) are reported in ppm and *J* values in Hz. Multiplicity is designated as doublet (d), double of double (dd), triplet (t), quartet (q) and multiplet (m). ^1^H NMR and ^13^C NMR chemical shifts are reported relative to d6-DMSO as an internal standard. The analysis of NMR spectra was made using MestReNova version 6.0.2 software. All commercial substances were purchased in Merck (Darmstadt, German).

*Synthesis of derivatives 2–5*

Quercetin **1** (0.33 mmol, 0.1 g) and potassium carbonate (5.5 eqv., 1.82 mmol, 0.251 g) were dissolved in 4 mL of dimethylformamide (DMF). The reaction mixture was heated to boiling temperature and the propyl or propene or propargyl bromide (5.5 eqv., 1.82 mmol) was added dropwise. The reaction progress was monitored by the thin layer chromatography (TLC) method. After substrate disappearance on the thin layer chromatography (TLC) plate, the reaction mixture was dissolved in water and extracted with chloroform (5 mL). The crude product was purified using column chromatography (SiO_2_, chloroform/ethanol, 40:1, v/v) method and give pure compounds **2–5**:

2-(3,4-dipropoxyphenyl)-5-hydroxy-3,7-dipropoxy-4*H*-chromen-4-one **2** yield 53%, m.p. 96–97 °C. ^1^H NMR (600 MHz, d6-DMSO) *δ* 0.91 (t, 3H, *J* = 7.8 Hz, CH_3_), 1.00 (m, 9H, 3 × CH_3_), 1.77 (m, 2H, CH_2_), 1.76 (m, 6H, 3 × CH_2_), 3.95 (t, 2H, *J* = 6.6 Hz, OCH_2_), 4.01 (t, 2H, *J* = 6.0 Hz, OCH_2_), 4.10 (m, 4H, 2 × OCH_2_), 6.37 (d, 1H, *J* = 2.4 Hz, H8), 6.78 (d, 1H, *J* = 2.4 Hz, H6), 7.16 (d, 1H, *J* = 9.0 Hz, H2’), 7.71 (m, 2H, H5’, H6’), 12.66 (s, 1H, OH); ^13^C NMR (150 MHz, d6-DMSO) *δ* 10.7, 10.8,10.9, 22.3, 22.5, 22.6, 23.4, 70.2, 70.4, 70.6, 74.2, 93.3, 98.5, 105.6, 113.3, 114.1, 122.8, 137.8, 148.4, 151.6, 156.1, 156.8, 161.4, 162.8, 165.1, 178.6. IR (ν_max_ cm^−1^, ATR): 2965–2875, 1662, 1587, 1501, 1316; HR-ESI (m/z) [M + H^+^] calculated for C_27_H_35_O_7_ 471.2383. Found: 471.2360.

3,7-bis(allyloxy)-2-(3,4-bis(allyloxy)phenyl)- 5-hydroxy-4*H*-chromen-4-one **3** yield 68%, m.p. 66–67 °C. ^1^H NMR (600 MHz, d6-DMSO) *δ* 4.64 (d, 2H, *J* = 6.0 Hz, OCH_2_), 4.59 (d, 2H, *J* = 6.0 Hz, OCH_2_), 4.71 (m, 4H, 2 × OCH_2_), 5.3 (m, 8H, 4 × CH = CH_2_), 5.98 (m, 1H, CH = CH_2_), 6.08 (m, 3H, 3 × CH = CH_2_), 6.42 (d, 1H, *J* = 2.4 Hz, H8), 6.82 (d, 1H, *J* = 2.4 Hz, H6), 7.19 (d, 1H, *J* = 9.0 Hz, H2’), 7.74 (m, 2H, H5’, H6’), 12.61 (s, 1H, OH); ^13^C NMR (150 MHz, d6-DMSO) *δ* 69.3, 69.5, 69.7, 73.0, 93.7, 98.8, 105.7, 113.6, 114.12, 118.1, 118.2, 118.6, 118.8, 122.8, 133.3, 133.8, 134.1, 134.2, 137.3, 147.8, 151.0, 156.1, 156.7, 161.4, 164.5, 178.6. IR (ν_max_ cm^−1^, ATR): 3090, 2923-2845, 1662, 1586, 1498, 1316; HR-ESI (m/z) [M + H^+^] calculated for C_27_H_27_O_7_ 463.1757. Found: 463.1710.

2-(3,4-bis(prop-2-yn-1-yloxy)phenyl)-5-hydroxy-3,7- bis(prop-2-yn-1-yloxy)-4H-chromen-4-one **4** yield 35%, m.p. 150–151 °C. ^1^H NMR (600 MHz, d6-DMSO) *δ* 3.54 (t, 1H, *J* = 2.4 Hz, C≡CH), 3.66 (m, 2H, 2 × C≡CH), 3.70 (t, 1H, *J* = 2.4 Hz, C≡CH), 4.90 (d, 2H, *J* = 2.4 Hz, OCH_2_), 4.97 (m, 6H, 3 × OCH_2_), 6.50 (d, 1H, *J* = 2.4 Hz, H8), 6.85 (d, 1H, *J* = 2.4 Hz, H6), 7.28 (d, 1H, *J* = 9.0 Hz, H2’), 7.81 (dd, *J*_*1*_ = 2.4 Hz, *J*_*2*_ = 9.0 Hz, 1H, H6’), 7.88 (d, *J* = 2.4 Hz,1H, H5’), 12.47 (s, 1H, OH); ^13^C NMR (150 MHz, d6-DMSO) *δ* 56.6, 56.8, 56.9, 59.6, 78.8, 79.0, 79.2, 79.3, 79.4, 79.6, 80.1, 94.2, 99.0, 105.9, 114.0, 115.0, 123.2, 123.4, 136.2, 146.8, 150.0, 156.5, 156.6, 161.4, 163.6, 178.5. IR (ν_max_ cm^−1^, ATR): 3288-3265, 2941-2875, 2123, 1650, 1593, 1497, 1302; HR-ESI (m/z) [M + H^+^] calculated for C_27_H_19_O_7_ 455.1131. Found: 455.1097.

2-(3,4-bis(prop-2-yn-1-yloxy)phenyl)-3,5,7- tris(prop-2-yn-1-yloxy)-4*H*-chromen-4-one **5** yield 48%, m.p. 91–92 °C. ^1^H NMR (600 MHz, d6-DMSO) *δ* 3.48 (t, 1H, *J* = 2.4 Hz, C≡CH), 3.64 (m, 3H, 3 × C≡CH), 3.65 (t, 1H, *J* = 2.4 Hz, C≡CH), 4.89 (d, 2H, *J* = 2.4 Hz, OCH_2_), 4.94 (m, 6H, 3 × OCH_2_), 4.94 (d, 2H, *J* = 2.4 Hz, OCH_2_), 6.65 (d, 1H, *J* = 2.4 Hz, H8), 6.95 (d, 1H, *J* = 2.4 Hz, H6), 7.26 (d, 1H, *J* = 9.0 Hz, H2’), 7.77 (dd, *J*_*1*_ = 2.4 Hz, *J*_*2*_ = 9.0 Hz, 1H, H6’), 7.87 (d, *J* = 2.4 Hz,1H, H5’); ^13^C NMR (150 MHz, d6-DMSO) *δ* 56.5, 56.8, 56.9, 57.0, 58.9, 78.8, 79.0, 79.3, 79.4, 79.5, 79.6, 79.7, 95.3, 98.9, 109.4, 114.0, 114.8, 122.8, 123.6, 138.1, 146.8, 149.6, 153.0, 158.3, 158.4, 161.8, 172.5. IR (ν_max_ cm^−1^, ATR): 3275–3211, 2947–2865, 2129, 2122, 1619, 1601, 1510-1488, 1298; HR-ESI (m/z) [M + H^+^] calculated for C_30_H_21_O_7_ 493.1287. Found: 493.1277.

### Determination of thermal stability (TGA, c-DTA)

The thermal stability of quercetin and tested derivatives (compound 2–5) was determined using thermogravimetric analysis (TGA). A TG 209 F3 Tarsus thermogravimeter (Netzsch, Germany) was employed for the measurements. Dynamic thermogravimetric analyses were carried out for all tested samples.

For each measurement, approximately 5 mg of sample was heated from 35 to 600 °C at a constant heating rate of 10 K min^−1^ under a nitrogen atmosphere. The total nitrogen flow rate was maintained at 40 ml min^−1^. An Al_2_O_3_ crucible was used for all measurements.

During the dynamic measurements, TG, DTG, and calculated DTA (c-DTA) curves were recorded. The c-DTA method was used to determine both endothermic and exothermic effects. Multiple-point temperature calibration was performed using c-DTA, based on the onset temperatures of melting peaks of high-purity reference materials (In, Sn, Zn, Al, BaCO_3_, and Au) covering the entire temperature range.

All thermogravimetric data were analyzed using Proteus 8.0 software (Netzsch, Germany).

### Assessment of DPPH radical scavenging activity

The antioxidant properties of quercetin and the tested derivatives were measured using a UV–Vis Genesys 10S spectrophotometer (Thermo Scientific, MA, USA). DPPH molecules (2,2-diphenyl-1-picrylhydrazyl) containing an unpaired electron were used as a reference for free radicals^29,30^.

A DPPH solution (0.5 mM in 96% ethanol) was prepared, to which the tested pure samples were added. For all measurements, 1 mg of each pure sample was added to 3 ml of the DPPH standard solution and mixed for 30 s. The DPPH standard solution and the DPPH solutions containing the tested samples were sequentially placed in polystyrene cuvettes with an optical path length of 1 cm, designed for spectrophotometric measurements.

The absorbance spectra of the DPPH standard solution and of the DPPH radicals in contact with the tested samples were recorded. Changes in the absorbance of DPPH radicals over time, corresponding to their interaction with the tested samples, were determined. The change in absorbance at a wavelength of 517 nm was analyzed, and spectra were recorded in the wavelength range of 450–600 nm. Measurements were performed using the VISIONlite spectroscopic software (Thermo Scientific, MA, USA).

For all samples, the kinetics of interaction with DPPH were studied. Measurements were taken at 5-min intervals up to 30 min, with an additional measurement after 1 min of reaction. All measurements were performed five times, and the results were averaged.

### Cell culture

In this study, three human ovarian adenocarcinoma cell lines: SKOV-3, OVCAR-3, and SW-626, obtained from the American Type Culture Collection (ATCC), as well as normal human fibroblasts (NHDF), were used. SKOV-3 cells were cultured in McCoy’s 5A modified medium supplemented with 10% fetal bovine serum (FBS) and antibiotics (100 U/mL penicillin and 100 µg/mL streptomycin). OVCAR-3 cells were maintained in RPMI-1640 medium supplemented with 20% FBS, 0.01 mg/mL insulin, and antibiotics. NHDF cells were cultured in DMEM supplemented with 10% FBS and antibiotics. All cells were maintained at 37 °C in a humidified atmosphere containing 5% CO_2_. SW-626 cells were cultured in Leibovitz’s L-15 medium, also supplemented with 10% FBS and antibiotics (100 U/mL penicillin and 100 µg/mL streptomycin), and maintained at 37 °C.

### MTT assay

To assess the cytotoxicity of the tested compounds, the MTT assay (3-[4,5-dimethylthiazol-2-yl]-2,5-diphenyltetrazolium bromide) was performed. Ovarian cancer cell lines SW626, OVCAR-3, and SKOV3, along with NHDF control cells, were seeded (20,000 cells per well for SW626, and 15,000 cells per well for NHDF, SKOV3, and OVCAR-3) in 96-well plates and incubated for 24 h at 37 °C in a 5% CO_2_ atmosphere in medium supplemented with 10% fetal bovine serum (FBS). After incubation, the medium containing the tested compounds at appropriate concentrations was added. The final DMSO concentration varied proportionally with compound concentration and ranged from 0.03125 to 0.5% across the tested dose range (6.25–100 µg/mL). The cells were then incubated for an additional 24 h, after which MTT reagent was added to a final concentration of 1 mg/mL. Cells were incubated for one more hour to allow for formazan crystal formation. Following incubation, the medium was removed, and the formazan crystals were dissolved in DMSO. The resulting solutions were transferred to new 96-well plates to minimize interference from residual cells. Absorbance was measured at 570 nm with background correction at 670 nm. The MTT assay was performed in three independent biological experiments. Each condition was tested in triplicate wells within each experiment.

### Flow cytometry analyses

The initial stages of sample preparation were analogous to those described above. After washing the cells twice (with PBS and Stain Buffer), the cells were centrifuged at 1000 rpm for 10 min, and the supernatant was then removed. The cell pellet was loosened by vortexing while slowly adding 5 mL of cold 70% ethanol, after which the samples were incubated overnight at –20 °C. After this period, the cells were washed twice to remove the ethanol. The cells were then centrifuged again for 10 min at 1000 rpm, and the supernatant was aspirated.

For cell staining, 0.5 mL of PI/RNase Staining Buffer (BD Pharmingen, San Diego, CA, USA), containing propidium iodide and RNase, was added to each sample. The cells were incubated with the dye for 15 min at room temperature. The samples were stored at 4 °C, protected from light, until analysis.

The stained cells were analyzed using a BD Accuri C6 Plus Flow Cytometer to determine the relative DNA content based on red fluorescence intensity. Each analysis was repeated three times. Flow cytometry analysis was performed in three independent biological experiments. In cytometric analysis of the cell cycle, 10,000 events corresponding to cellular singlets were collected for each sample. For cell cycle analysis, 10,000 events corresponding to cellular singlets were collected for each sample.

Data analysis was performed using Kaluza software.

In the first step, cellular debris was excluded based on FSC-A vs. SSC-A parameters by gating the population of cells with normal size and granularity. Next, aggregates and doublets were eliminated by analyzing PI-A vs. PI-H, selecting the population of single cells.

Cell cycle analysis was conducted within the single-cell gate using a histogram of PI fluorescence intensity, determining the proportion of cells in G0/G1, S, and G2/M phases. Additionally, the sub-G1 fraction was analyzed as an indicator of DNA fragmentation.

Statistical analysis of the results was performed using two-way analysis of variance (Two-way ANOVA) along with Dunnett’s multiple comparisons test, using GraphPad Prism software. Differences were considered statistically significant at *p* < 0.05 (Fig. 1).

## Results and discussion

### Chemical synthesis

**Fig. 2:**
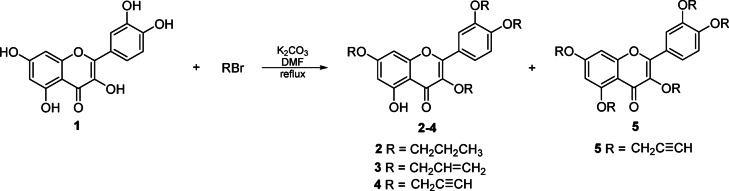
Synthetic route to compounds 2–5.

The alkoxy derivatives of quercetin **2–5** obtained in the reaction between quercetin **1** and propyl, allyl or propargyl bromide in the presence of potassium carbonate and dimethylformamide (Fig. 2).

After purification of column chromatography obtain the pure products with yield 35–68% In the reaction with ally and propyl bromide obtained only one product **2–3**, while propargyl bromide two compounds **4** and **5**. The chemical structure of derivatives **2–5** determined by HR-MS, ^1^H and ^13^C NMR and IR spectroscopy.

### Thermal analysis

#### Thermogravimetric analysis (TG/DTG/D2TG)

**Fig. 3:**
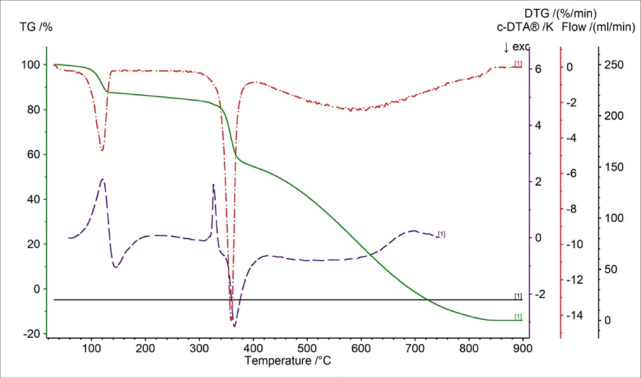
Thermogravimetric curve (green line), first derivative of the thermogravimetric curve (red line), and differential thermal analysis curve (purple line) recorded for quercetin. The measurement was carried out in a nitrogen atmosphere.

**Fig. 4:**
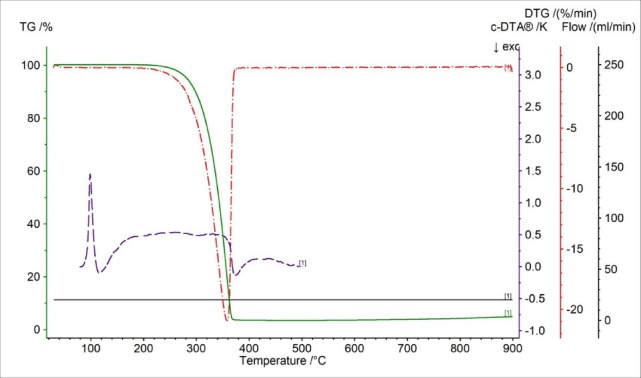
Thermogravimetric curve (green line), first derivative of the thermogravimetric curve (red line), and differential thermal analysis curve (purple line) recorded for compound **2**. The measurement was carried out in a nitrogen atmosphere.

**Fig. 5:**
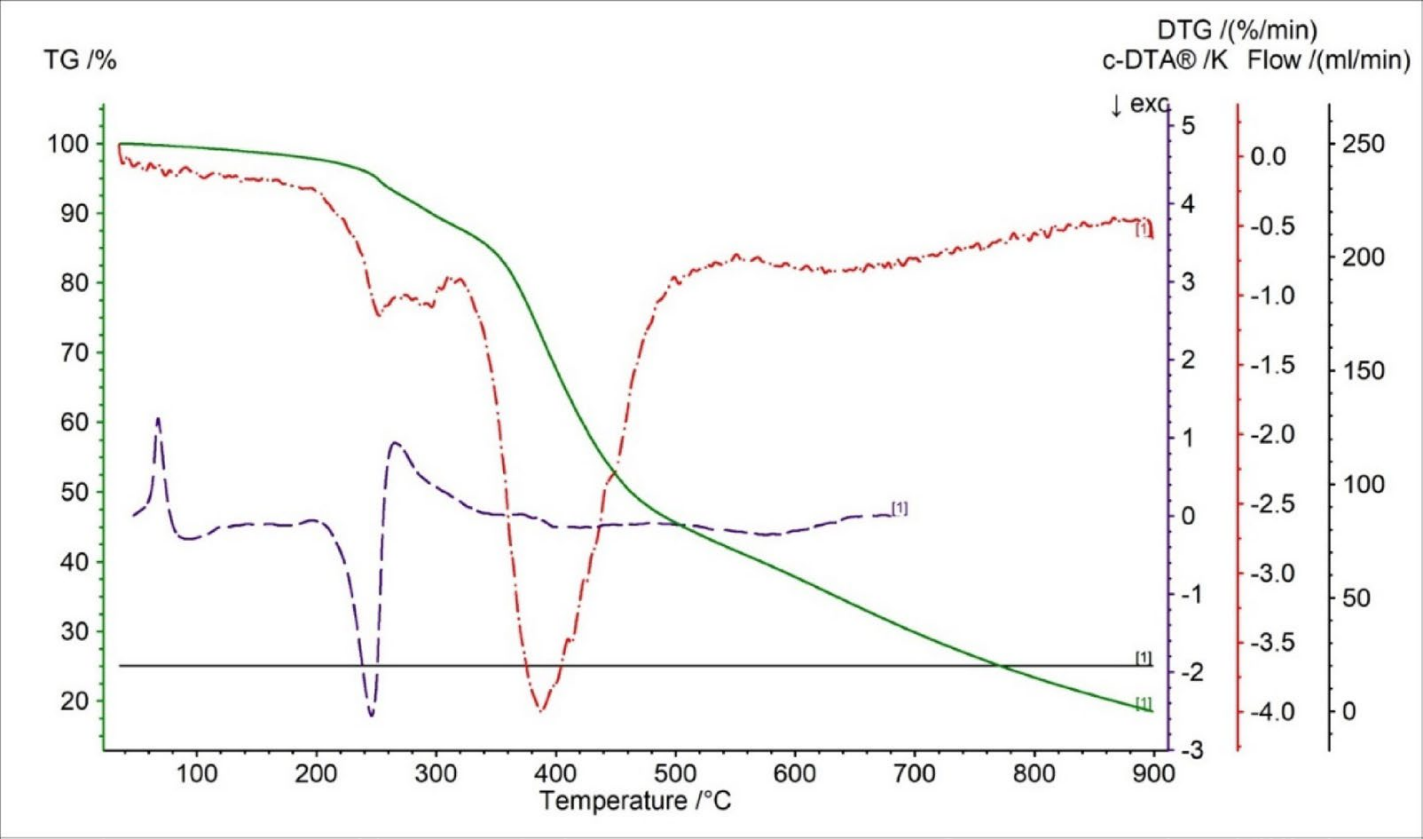
Thermogravimetric curve (green line), first derivative of the thermogravimetric curve (red line), and differential thermal analysis curve (purple line) recorded for compound **3**. The measurement was carried out in a nitrogen atmosphere.

**Fig. 6:**
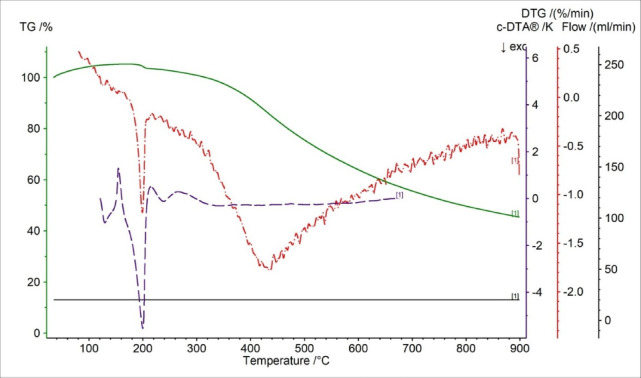
Thermogravimetric curve (green line), first derivative of the thermogravimetric curve (red line), and differential thermal analysis curve (purple line) recorded for compound **4**. The measurement was carried out in a nitrogen atmosphere.

**Fig. 7:**
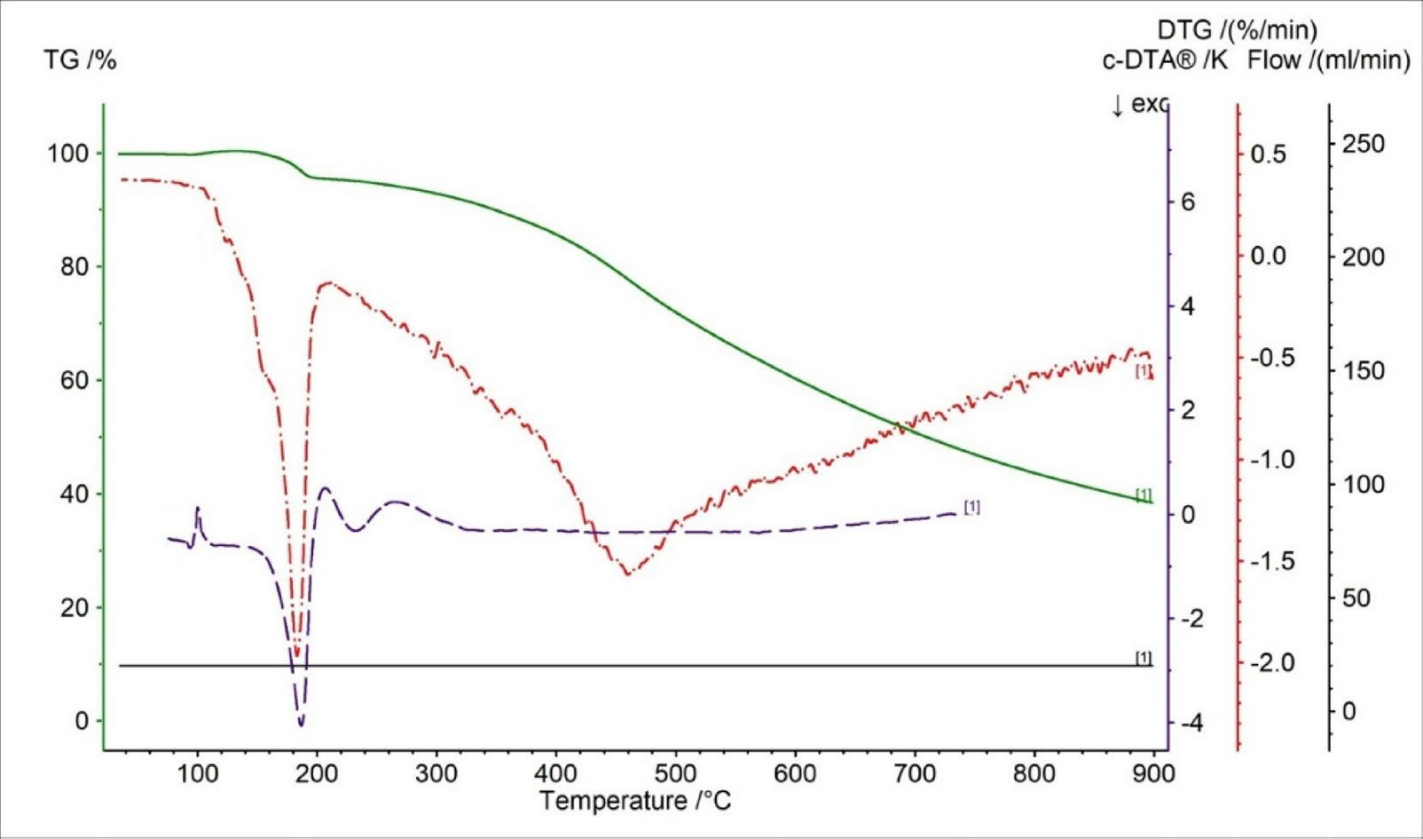
Thermogravimetric curve (green line), first derivative of the thermogravimetric curve (red line), and differential thermal analysis curve (purple line) recorded for compound **5**. The measurement was carried out in a nitrogen atmosphere.

Thermogravimetric analysis (TG), together with its first (DTG) and second (D2TG) derivative curves, was used to evaluate the thermal stability and decomposition behavior of the newly synthesized compounds compound **2–5** in comparison with quercetin (reference compound). The results are presented in Figs. 3, 4, 5, 6, 7 and Tables 1, 2 and 3.

**Table 1:** Thermogravimetric parameters of quercetin and the tested derivatives.

Samples	Onset [°C]	Mid [°C]	Infection [°C]	End [°C]	Mass change [%]
1	344.4	351.4	359.1	361.0	− 16.12
2	319.2	341.8	357.4	366.5	− 95.48
3	339.7	392.6	388.0	451.0	− 36.20
4	320.5	408.7	460.9	566.9	− 31.25
5	178.7	438.3	459.5	569.6	− 32.31

**Table 2:** DTG curve parameters for quercetin and the tested derivatives.

Samples	STAGE 1		STAGE 2		STAGE 3		STAGE 4	
	[°C]	%/min	[°C]	%/min	[°C]	%/min	[°C]	%/min
1	119.7	− 4.77	359.0	− 15.73	581.1	− 2.66	–	–
2	357.6	− 21.0	–	–	–	–	–	–
3	251.4	− 1.16	296.9	− 1.11	387.8	− 4.02	625.0	− 0.87
4	198.6	− 1.34	438.6	− 1.82	–	–	–	–
5	183.4	− 0.2	459.4	− 0.1	–	–	–	–

**Table 3:** D2TG curve parameters for quercetin and the tested derivatives.

Samples	STAGE 1		STAGE 2		STAGE 3		STAGE 4	
	Begin [°C]	End [°C]	Begin [°C]	End [°C]	Begin [°C]	End [°C]	Begin [°C]	End [°C]
1	110	126	352	364	500	680	–	–
2	340	366	–	–	–	–	–	–
3	243	262	282	299	360	429	620	680
4	195	202	324	538	–	–	–	–
5	179	189	313	508	–	–	–	–

The TG curve for quercetin **1** showed that the thermal decomposition of the compound begins at 344.4 °C (Fig. 3, Table 1). The DTG and D2TG curves indicate three transformation stages. The first stage, with a maximum at approximately 120 °C, is associated with the loss of moisture and small molecules (Fig. 3, Tables 2, 3). The second stage, with maximum mass loss at around 360 °C, corresponds to the main decomposition of the flavonol structure. The final stage, recorded on the DTG curve with a mass-loss maximum at 581.1 °C, has a broad shape and is associated with the carbonization of organic residues (Fig. 3, Table 2). All these stages correlate with literature data concerning the thermal decomposition of quercetin^31,32^.

For the newly synthesized compounds, distinct differences in decomposition behavior were observed. Compound **2** underwent rapid single-step degradation, with a DTG maximum at 357.6 °C, accompanied by nearly complete mass loss (−95.5%), indicating low thermal resistance and total structural decomposition (Fig. 4, Tables 1, 2, 3).

Compound **3** decomposed in four stages, with DTG maxima at 251.4 °C, 296.9 °C, 387.8 °C, and 625 °C (Fig. 5, Tables 1, 2, 3), suggesting a complex process involving gradual decomposition of functional groups. The onset temperature of compound **3** decomposition is the closest to that of quercetin among all new compounds (Fig. 5, Table 1).

Compound **4** exhibited a two-step decomposition pattern, with a decomposition onset at 320.5 °C (Fig. 6, Table 1). The DTG curve shows two mass-loss maxima at 198.6 °C and 438.6 °C (Fig. 6, Tables 2, 3), indicating moderate thermal stability.

The least thermally stable compound among those tested was compound **5**, whose decomposition began at approximately 180 °C (Fig. 7, Table 1). This compound underwent a two-step thermal degradation similar to compound 4. The DTG curve shows maxima at 183.4 °C and 459.4 °C (Fig. 7, Tables 2, 3).

Comparison of decomposition onset temperatures indicates that the thermal stability of the tested compounds increases in the following order: **5** < **2** < **4** < **3** < **1**.

The results confirm that substitution within the quercetin structure leads to significant changes in the thermal decomposition process, which may be related to the presence of different functional groups and varying degrees of electronic conjugation in the aromatic system. Compounds **2–5** all exhibit lower thermal resistance compared to the reference compound. Among them, compound **5** shows the lowest stability, while compound **3** displays the highest, comparable to QUE.

Thermogravimetric analysis (TGA) provided information on the thermal stability and decomposition profile of the tested compounds. Knowledge of thermal stability is important for the storage, handling, and formulation of these compounds for biological applications. Although TGA does not directly measure anticancer activity, ensuring compound stability under experimental conditions supports the reliability of cytotoxicity results and the reproducibility of biological assays.

#### Differential thermal analysis (c-DTA)

The differential thermal analysis (c-DTA) curves provided additional information on the nature of the observed transformations. For quercetin, two endothermic effects were observed (approx. 120 °C and 326 °C), corresponding to the evaporation of crystallization water and melting point of the substance, respectively, followed by two exothermic effects at 364.7 °C and 497 °C, attributed to oxidation and carbonization of the organic residue (Fig. 2, Table 4)^31,32^.

**Table 4:** Differential thermal analysis (c-DTA) curves for quercetin and the tested derivatives.

Samples	Stage	Onset [°C]	Peak [°C]	Area [K*s]	Reaction
1	1	96.9	120.6	466.97	ENDOTHERMIC
	**2**	**324.0**	**326.1**	**107.51**	**ENDOTHERMIC**
	3	354.8	364.7	333.33	EXOTHERMIC
	4	–	497.0	609.94	EXOTHERMIC
2	**1**	**94.5**	**98.3**	**82.22**	**ENDOTHERMIC**
	2	364.4	372.8	93.30	EXOTHERMIC
3	**1**	**62.8**	**67.6**	**87.90**	**ENDOTHERMIC**
	2	223.6	246.1	40.35	EXOTHERMIC
	3	272.2	360.3	100.47	EXOTHERMIC
	4	–	430.7	39.51	EXOTHERMIC
	5	–	575.8	98.82	EXOTHERMIC
4	**1**	**151.6**	**154.4**	**103.31**	**ENDOTHERMIC**
	2	184.2	200.0	460.85	EXOTHERMIC
	3	220.8	235.6	74.80	EXOTHERMIC
	4	–	360.6	423.87	EXOTHERMIC
5	**1**	**82.4**	**91.6**	**17.20**	**ENDOTHERMIC**
	2	168.3	186.5	268.80	EXOTHERMIC
	3	215.8	233.2	113.22	EXOTHERMIC
	4	–	338.2	960.93	EXOTHERMIC

Melting points are shown in bold.

For compound **2** an endothermic effect was recorded at 98.3 °C, corresponding to its melting point, and a strong exothermic peak at 372.8 °C, related to rapid degradation and combustion of the compound (Fig. 3, Table 4).

Compound **3** showed a more complex thermal profile, including one endothermic effect associated with melting point (67.6 °C) and several subsequent exothermic events in the range of 246–576 °C, confirming a multistage decomposition mechanism (Fig. 4, Table 4).

Compound **4** exhibited one endothermic effect corresponding to melting point (154.4 °C) and three distinct exothermic peaks at 200 °C, 236.6 °C, and 360.6 °C, corresponding to successive oxidation steps of intermediate products (Fig. 5, Table 4).

In contrast, compound **5** displayed a weak endothermic effect at 91.6 °C (melting point) and strong exothermic transformations in the range 186–338 °C, with a large surface area of 960.93 K·s (stage 4), suggesting an intense exothermic reaction—likely oxidation and structural reorganization of the sample (Fig. 6, Table 4).

It should be emphasized that all melting peaks have sharp shapes with well-defined maxima, which, according to the scientific literature, indicates high purity of both quercetin and all synthesized derivatives^33^ (Tables 5, 6).

**Table 5:** The IC_50_ (µM) and R^2^ values for compound 2–4 in SKOV3 and SW626 cell lines.

Compound	Cell line	IC_50_	R^2^
2	SKOV3	98.29	0.6056
	SW626	1.853	0.8980
	OVCAR 3	> 100	–
3	SKOV3	> 100	–
	**SW626**	**8.155**	**0.7993**
	OVCAR 3	**6.632**	**0.9763**
4	SKOV3	> 100	–
	SW626	18.03	0.7993
	OVCAR 3	15.40	0.8473

IC_50_ values were calculated using nonlinear regression with a four-parameter logistic model in GraphPad Prism. Significant values are in bold.

**Table 6:** Effect of compounds 2–5 and quercetin on the proliferation of SW626 (a), SKOV3 (b), and OVCAR3 (c) cells relative to NHDF cells, as determined by two-way ANOVA (factors: compound and concentration) followed by Tukey’s multiple comparisons test.

NHDF						
	Compound	Q	2	3	4	5
	µg/ml					
SW626	(a)					
	0	NS	NS	NS	NS	NS
	6,25	NS	< 0.001	< 0.001	NS	NS
	12,5	NS	< 0.001	< 0.001	< 0.001	NS
	25	< 0.01	< 0.001	< 0.001	< 0.001	NS
	50	< 0.01	< 0.001	< 0.001	< 0.001	NS
	100	NS	< 0.001	< 0.001	< 0.001	NS
SKOV3	(b)					
	0	NS	NS	NS	NS	NS
	6,25	< 0.05	NS	< 0.01	NS	NS
	12,5	< 0.05	< 0.001	NS	NS	< 0.05
	25	< 0.05	NS	< 0.01	NS	< 0.01
	50	< 0.05	NS	NS	NS	< 0.01
	100	< 0.05	NS	< 0.05	< 0.01	NS
OVCAR 3	(c)					
	0	NS	NS	NS	NS	NS
	6,25	NS	< 0.05	< 0.001	< 0.001	< 0.001
	12,5	NS	< 0.001	< 0.001	< 0.001	< 0.001
	25	NS	NS	< 0.001	< 0.001	< 0.01
	50	< 0.01	< 0.01	< 0.001	< 0.001	NS
	100	NS	< 0.001	< 0.001	< 0.001	NS

Data are presented as mean ± SD. Statistical significance was defined as NS (not significant), *p* < 0.05, *p* < 0.01, *p* < 0.001.

### Analysis of antioxidant properties using the DPPH method

The antioxidant activity of the tested compounds was evaluated using the 2,2-diphenyl-1-picrylhydrazyl (DPPH) radical assay, which measures the ability of a sample to neutralize free radicals based on the colour change of the DPPH solution from violet to yellow^29,30^. The intensity of this phenomenon, measured as a decrease in absorbance at 517 nm, reflects the strength of the antioxidant activity. Quercetin—a well-known natural antioxidant—was used as the reference compound.

#### Absolute activity

**Fig. 8:**
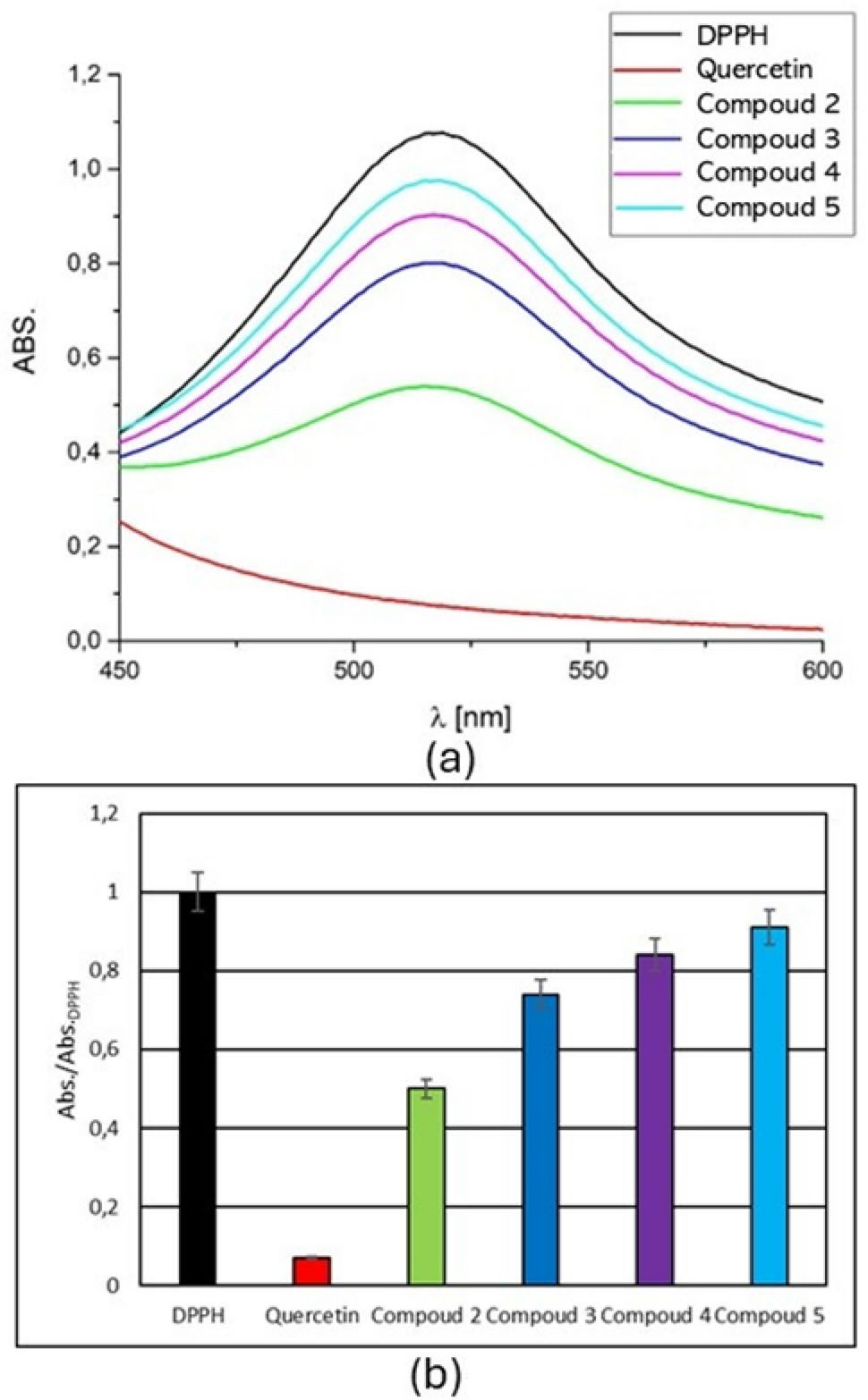
(**a**) Absorbance spectra of initial DPPH (black line) and DPPH after 30 min of interaction with the tested samples. (**b**) Absorbance values after 30 min of interaction with the tested samples. Measurements were performed at 517 nm.

A comparison of the activities of derivatives **2–5** with that of quercetin showed that all newly synthesized compounds exhibit antioxidant activity, albeit to different extents (Fig. 8). As expected, quercetin displayed the highest DPPH radical scavenging ability, reaching the maximum inhibition percentage shortly after the reaction began^34^.

Among the tested derivatives, compound **2** demonstrated the highest activity. The derivatives **3–4** showed moderate antioxidant activity, whereas compound **5** exhibited the lowest radical scavenging ability, which is associated with the absence of hydroxyl groups responsible for proton donation.

#### Reaction kinetics

**Fig. 9:**
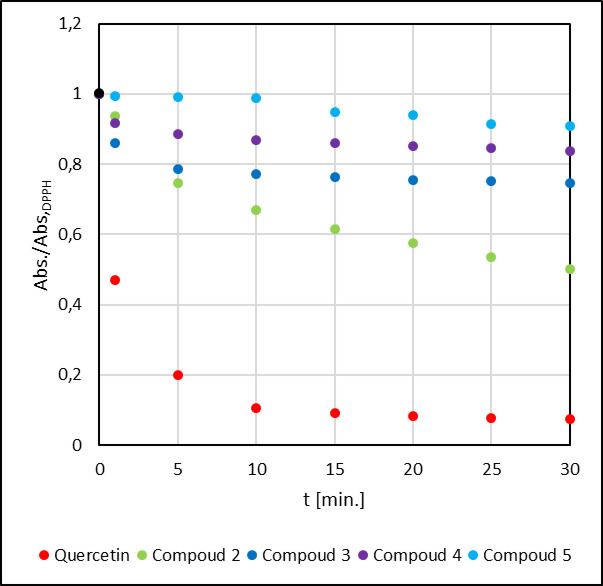
Kinetics of interaction of quercetin and the tested derivatives with DPPH free radicals. Change in absorbance measured at a wavelength of 517 nm.

Kinetic analysis of the DPPH reaction with the tested compounds indicates that the reduction of the radical by quercetin proceeds very rapidly—a sharp decrease in absorbance is observed within the first few minutes, characteristic of strong antioxidants with high electron reactivity (Fig. 9)^34^.

Compounds **3** and **4** showed a similar but slightly slower reaction profile, suggesting that their mechanism involves the gradual transfer of hydrogen atoms from a smaller number of reactive –OH groups compared to the flavonol system of quercetin. The derivative **5** exhibited the slowest reaction course, with a gradual and minimal decrease in DPPH absorbance, indicating weak electron-donating ability. Among the tested samples, the compound 2 showed the fastest DPPH radical quenching, demonstrating its high capacity for rapid electron donation.

The obtained results suggest that substitutions in the quercetin molecule affect both the number and the spatial distribution of hydroxyl groups, which directly determine its ability to transfer electrons and stabilize the resulting radicals. Moreover, the studies confirmed that derivatives containing substituents with double or triple bonds exhibit weaker interactions with the DPPH radical compared to those with single-bonded substituents.

### MTT test

**Fig. 10:**
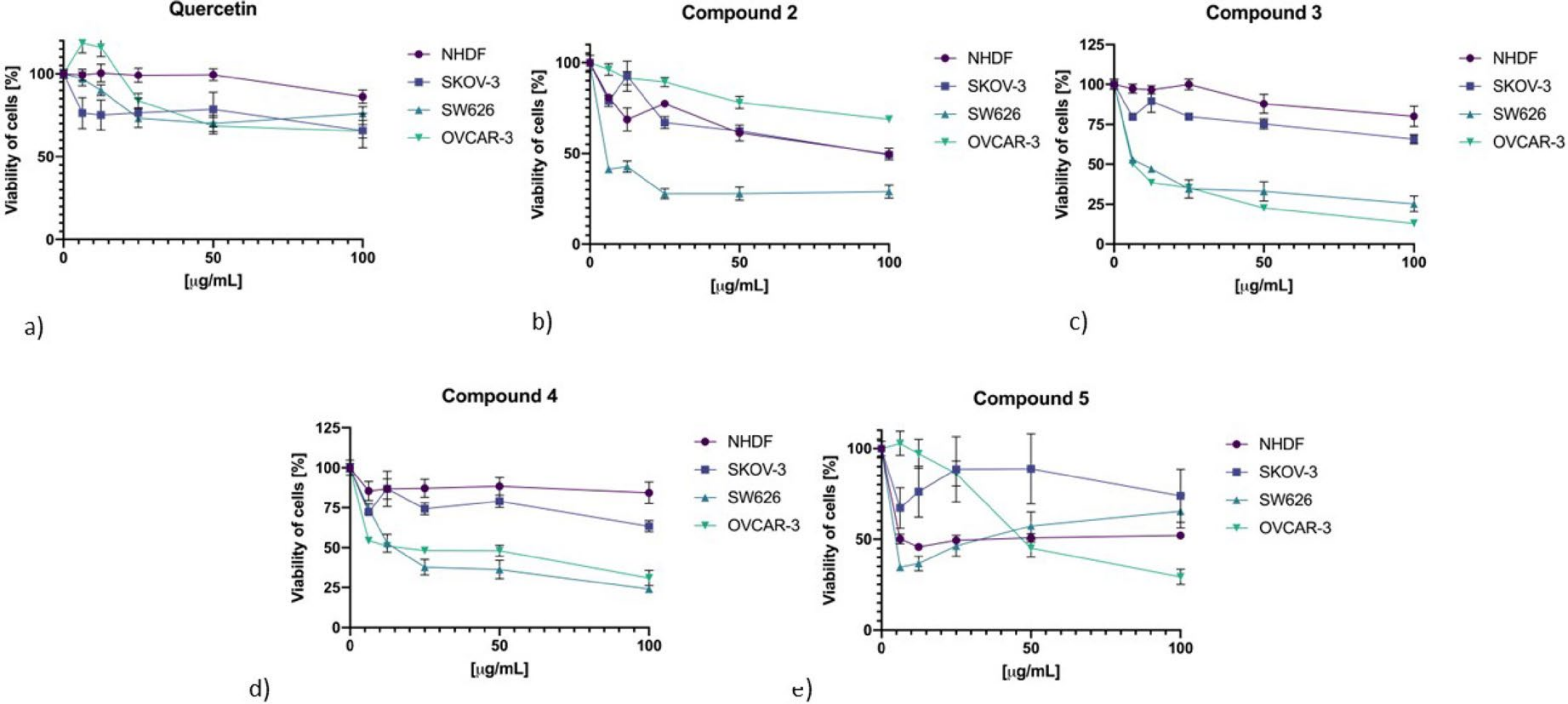
Effect of quercetin and derivatives 2–5 on the proliferation of SKOV3, SW626, OVCAR3, and NHDF cells (MTT assay).

The effect of quercetin 2–5 derivatives and quercetin itself on the proliferation of SW626, SKOV3, and OVCAR 3 cancer cell lines, as well as NHDF control cells, was assessed using the MTT assay, which measures cell metabolic activity as an indicator of cell survival. The cells were incubated with different concentrations of the compounds (6.25 μg/mL, 12.5 μg/mL, 25 μg/mL, 50 μg/mL, 100 μg/mL), and then the percentage of cell viability was measured. The results are presented in graphs showing the relationship between the concentration of a given compound and the % viability for each line (Fig. 10). The results obtained showed a varied response of cells to the tested substances. Based on the data, IC_50_ values were calculated, which indicate the concentration necessary to inhibit proliferation by 50%.

Compound **2** showed very strong activity against SW626 cells (IC₀ > = 1.853, R^2^ = 0.8980) and moderate activity against SKOV3 cells (IC_50_ = 98.29, R^2^ = 0.6056). Its effect on the OVCAR3 line was weak (IC_50_ > 100). However, this compound also exhibited a strong cytotoxic effect on normal cells, comparable to its effect on cancer cells, indicating its low selectivity and potential toxicity.

Compound **3** showed clear activity against the SW626 line, achieving an IC_50_ value of 8.155 with R^2^ = 0.7993. Strong cytotoxicity was also observed against OVCAR3 cells (IC_50_ = 6.632, R^2^ = 0.9763). However, this compound showed little activity against the SKOV3 line (IC_50_ > 100), indicating potential selectivity towards SW626 and OVCAR3 cells.

Compound **4** showed moderate cytotoxicity against SW626 cells (IC_50_ = 18.03, R^2^ = 0.7993) and OVCAR3 cells (IC_50_ = 15.40, R^2^ = 0.8473) cells, as well as very low activity against SKOV3 cells (IC_50_ > 100), which may indicate a certain degree of selectivity towards the SW626 and OVCAR3 cell lines.

For compound **5**, it was not possible to reliably determine the IC_50_ value under the conditions used. Due to these limitations and the lack of preliminary indications of significant activity, this compound was not pursued further in the current study (Table 5).

Statistical analysis (ANOVA test) (Table 6) showed significant differences between cancer lines and control cells. It was found that:

Compounds 2–4 exhibited high cytotoxic activity against the SW626 cell line, compared to NHDF control cells, reducing proliferation in a concentration-dependent manner (Fig. 10b-d). In addition, Compound 4 showed high cytotoxic activity against the OVCAR 3 line, compared to NHDF control cells.

**Fig. 11:**
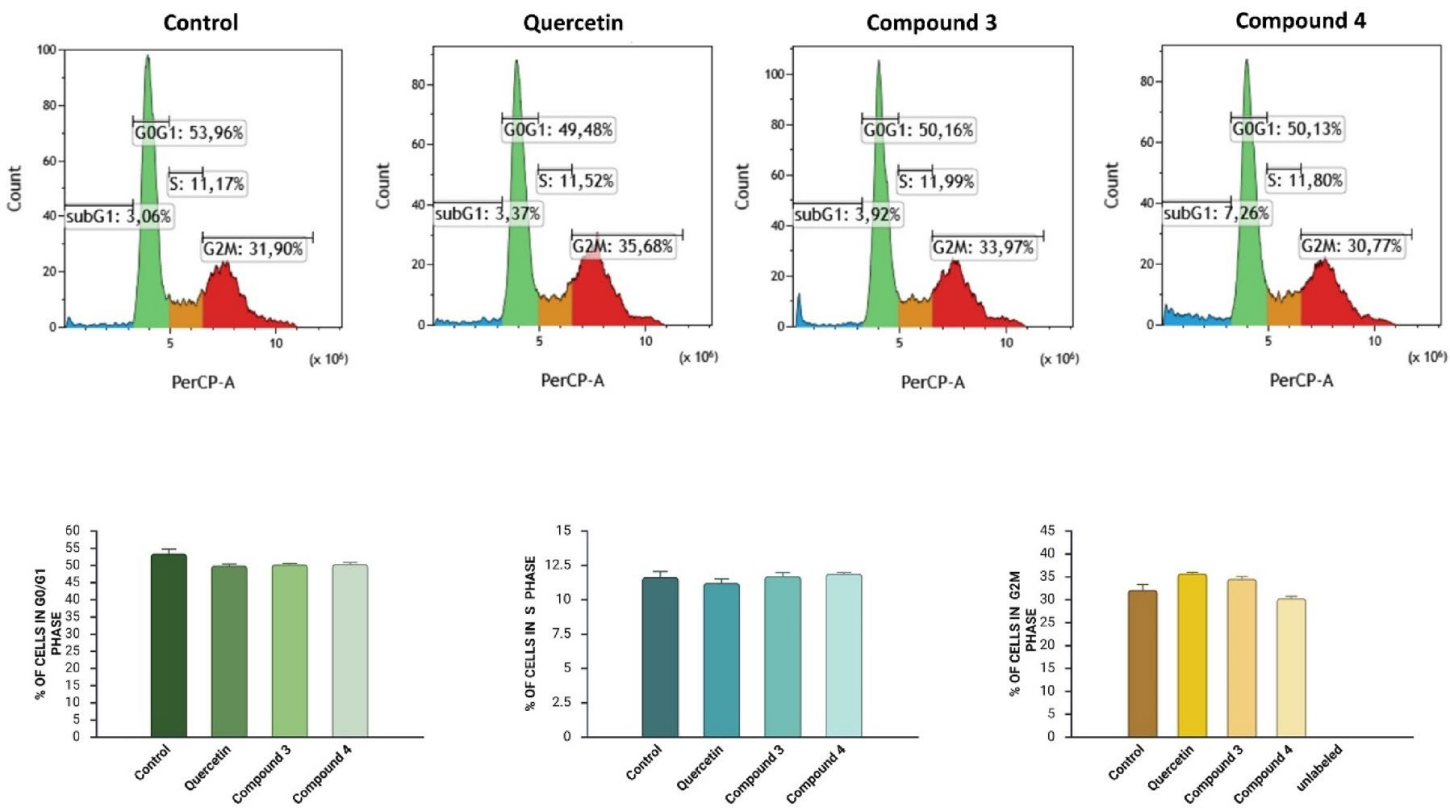
Effect of the tested compounds on the cell cycle. Bars represent the mean ± SD from three independent experiments (n = 3). No statistically significant differences were observed compared to the control group in any phase of the cell cycle (Two-way ANOVA, Dunnett’s test; *p* > 0.05).

Compound 3 showed inhibitory effects on proliferation, also in relation to the SKOV3 and OVCAR3 lines, suggesting that its chemical structure promotes cytotoxicity against cancer cells (Fig. 11).

Compound 5 showed the weakest effect on all cell lines compared to normal cells, consistent with previous observations regarding its limited antioxidant activity, and therefore it will not be considered for further analysis.

The initial toxicity of the compounds was assessed against normal NHDF cells. When comparing the effects of the compounds on this cell line, it was found that the control NHDF cells exposed to derivatives 3 and 4 were largely resistant to their effects, indicating a certain cytotoxic selectivity towards cancer cells.

In summary, among the group of compounds 2–5 studied, derivative 3 showed the most favorable activity profile—a moderately strong cytotoxic effect against SW636 and OVCAR3 cancer cells, with an acceptable dose–response curve fit and potential selectivity. Compound 4, despite its higher IC_50_ value, may also be an interesting subject for further research due to its weaker effect on other lines and potentially lower toxicity to normal cells. Compound 2, despite its high activity, was excluded from further analysis due to its strong activity against normal cells. As a result, two compounds were selected for further study: 3 and 4, which showed the most promising profile of biological activity and selectivity against cancer cells.

These observations are consistent with the literature, which indicates that modifications of quercetin enhance its anticancer activity, and that its effects on cancer cell proliferation depend on the type and position of substituents. Structural modifications, such as glycosylation, methylation, sulfation, and glucuronidation, combined with nanoparticle formulations, significantly improve the stability, solubility, and bioavailability of quercetin, enabling targeted drug delivery^35^.

Similar results were reported in a study investigating the impact of quercetin acetylation on its biological properties. The authors demonstrated that acetylation of the hydroxyl groups of quercetin (3, 7, 3′, 4′-OH) resulted in the formation of 3,7,3′,4′-O-tetraacetylquercetin (4Ac-Q), whose anticancer activity was significantly stronger compared to that of unmodified quercetin^36^. Other studies have evaluated the effects of acetyl and methyl modifications on quercetin activity. It was shown that the acetylated derivative 3,3′,4′,7-O-tetraacetylquercetin strongly inhibited cell proliferation and induced apoptosis in both MCF-7 and MDA-MB-231 breast cancer cells. Acetylation of the hydroxyl groups in the quercetin molecule significantly enhances its anticancer activity, bioavailability, and ability to induce apoptosis across various cancer cell types^37^. Interesting results were also obtained in studies on lipophilic quercetin derivatives, in which an n-pentyl substituent was introduced into the flavonoid molecule via an ether linkage to each hydroxyl group. This approach allowed the quercetin molecule to target cell membranes, increasing its local concentration and enhancing its effectiveness in interacting with membrane-associated signaling kinases^16^.

The obtained results, combined with literature data, confirm that the introduction of multiple bonds and modifications of the hydroxyl groups in the quercetin molecule significantly influence its anticancer activity. These changes can alter both the electronic and conformational properties of the molecule, thereby affecting its ability to interact with biological targets such as enzymes or cellular receptors.

These findings are consistent with our observations, in which alkoxylated quercetin derivatives inhibited cancer cell proliferation in a dose-dependent manner. Structure–activity relationship analysis revealed that the hydroxyl group at the C5 position significantly influences the anticancer activity of these compounds. Comparing the effects of compounds 2–4, it was observed that the introduction of multiple bonds enhances their activity against the tested cell lines.

However, it should be noted that the concentrations used in this study (6.25–100 µg/ml), while suitable for evaluating dose–response relationships in vitro, may not reflect physiologically achievable levels in vivo. Therefore, the observed cytotoxic effects of the tested compounds should be interpreted with caution. The MTT assay was selected as it enables the detection of early changes in cellular metabolic activity, allowing for the assessment of subtle, early-stage cytotoxic effects prior to the onset of membrane damage. However, the MTT assay employed here measures cellular metabolic activity rather than direct cell death, and thus some of the observed effects may partially reflect metabolic alterations induced by the compounds rather than actual cell death. It should also be noted that compound-only blanks without cells were not included in the MTT assay to control for potential absorbance interference from the colored quercetin derivatives, and therefore some contribution of the compounds intrinsic color to the measured absorbance cannot be ruled out. Although the DMSO concentration varied across dose points, its impact is expected to be minimal; however, the use of vehicle-matched controls for each concentration would further strengthen the interpretation of the results. Taken together, these limitations highlight the need for cautious interpretation of the results. Future studies will aim to validate these findings under physiologically relevant conditions and employ complementary approaches to assess cell viability and cytotoxicity.

### Flow cytometry analysis

The study evaluated the effect of Quercetin and two of its derivatives on cell division in specific phases of the cell cycle (G0/G1, S, G2/M) compared to the control (OVCAR 3 cells not exposed to the compounds). The analysis included three experimental replicates, and the values are presented as a percentage mean and standard deviation.

In the G0/G1 phase, all compounds tested caused a slight decrease in the percentage of cells compared to the control (from 53.32% in the control, QUE; 49.83 Compound 3; 50.18, Compound 4; 50.27). In the S phase, the percentage of cells remained unchanged under all conditions, indicating that the tested compounds do not significantly affect cell replication activity.

Flow cytometry analysis showed that incubation of the cells with the tested compounds did not disrupt cell cycle progression. Statistical analysis of the cell cycle was performed using a two-way ANOVA followed by Dunnett’s post hoc test. No statistically significant differences were observed between the control group and the tested samples (Control vs. Quercetin: *p* > 0.9999; Control vs. Compound 3: *p* > 0.9999; Control vs. Compound 4: *p* = 0.9996), indicating that the analyzed compounds had no effect on the distribution of cell cycle phases under the tested conditions.Figure 11 

Notably, while previous studies Azizi et al. reported G2/M arrest induced by quercetin, the differences may reflect variations in experimental conditions such as compound concentration, treatment duration, or cell line used. Importantly, the direction of the effect we observed for is consistent with the literature, although in our experiments it did not reach biological significance^38^. Similar results were obtained in studies on the effect of quercetin on colon cancer cells (HT29), after which cell cycle arrest occurred in the G2/M phase, slowing down their division^39^. In turn, studies conducted by Ren et al.^40^ involving SKOV 3 cells showed cycle arrest in the G0/G1 phase after the use of Que and a decrease in the percentage of cells in the G2/M phase^40^. In our study, we decided not to use SKOV-3 cells, even though previous studies have shown that quercetin can induce cycle arrest. The reason was the low sensitivity of SKOV-3 cells to the compound and its derivatives in the MTT test, in which we measured the cytotoxicity of the compound against cells. Preliminary tests showed that even at higher concentrations, quercetin and its derivatives had only a weak anti-proliferative effect, suggesting a limited effect of the compound on the cell cycle.nTherefore, in our study, we focused on the OVCAR 3 cell line, which was more sensitive to quercetin, allowing us to obtain more reliable results regarding the effects on the cell cycle.

## Conclusions

The results of TG, DTG, D2TG, and c-DTA analyses confirm that structural modifications of quercetin significantly influence thermal stability and decomposition behavior. Quercetin exhibits a predictable three-stage decomposition and good thermal resistance, whereas the newly synthesized compounds show a greater diversity of thermal transformations. Among the studied derivatives, **3** and **4** stand out for their higher thermal stability and the presence of multiple exothermic effects, which may indicate the formation of stable intermediates or highly cross-linked aromatic structures.

The obtained results for antioxidant properties suggest that substitutions in the quercetin molecule affect both the number and the spatial distribution of hydroxyl groups, which directly determine its ability to transfer electrons and stabilize the resulting radicals. Moreover, the studies confirmed that derivatives containing substituents with double or triple bonds exhibit weaker interactions with the DPPH radical compared to those with single-bonded substituents.

The MTT assay enabled the identification of quercetin derivatives that exhibit the strongest effects on cell metabolic activity, including compounds 3 and 4, in the tested cancer cell lines. However, it should be noted that the MTT assay reflects metabolic activity rather than direct cell death, therefore, the observed effects should be interpreted as preliminary indicators of cytotoxic or antiproliferative activity. Further studies are required to confirm these effects using complementary methods.

In summary, the obtained results, together with literature data, suggest that targeted structural modifications of quercetin, such as the introduction of multiple bonds and modifications of hydroxyl groups, may represent a promising strategy for modulating the biological activity of flavonoids. These findings provide a basis for further investigation of quercetin derivatives as potential candidates for future studies.

## Supplementary Information

Below is the link to the electronic supplementary material.

Supplementary Material 1

## Data Availability

The datasets used and/or analysed during the current study are available from the https://ppm.sum.edu.pl/info/researchdata/SUMc861ac31efde48e7a3b2b4d5cd2b8cb1/
