# Supplementary material for: Synthesis and application of quercetin hybrids as potential anticancer agents in the treatment of ovarian cancer
Source: Sci Rep. 2026 Apr 28;16:19752. doi: 10.1038/s41598-026-50954-9 (PMC13314938; doi:10.1038/s41598-026-50954-9)

Figure S1. The ^{1}H NMR spectrum of compound **2**

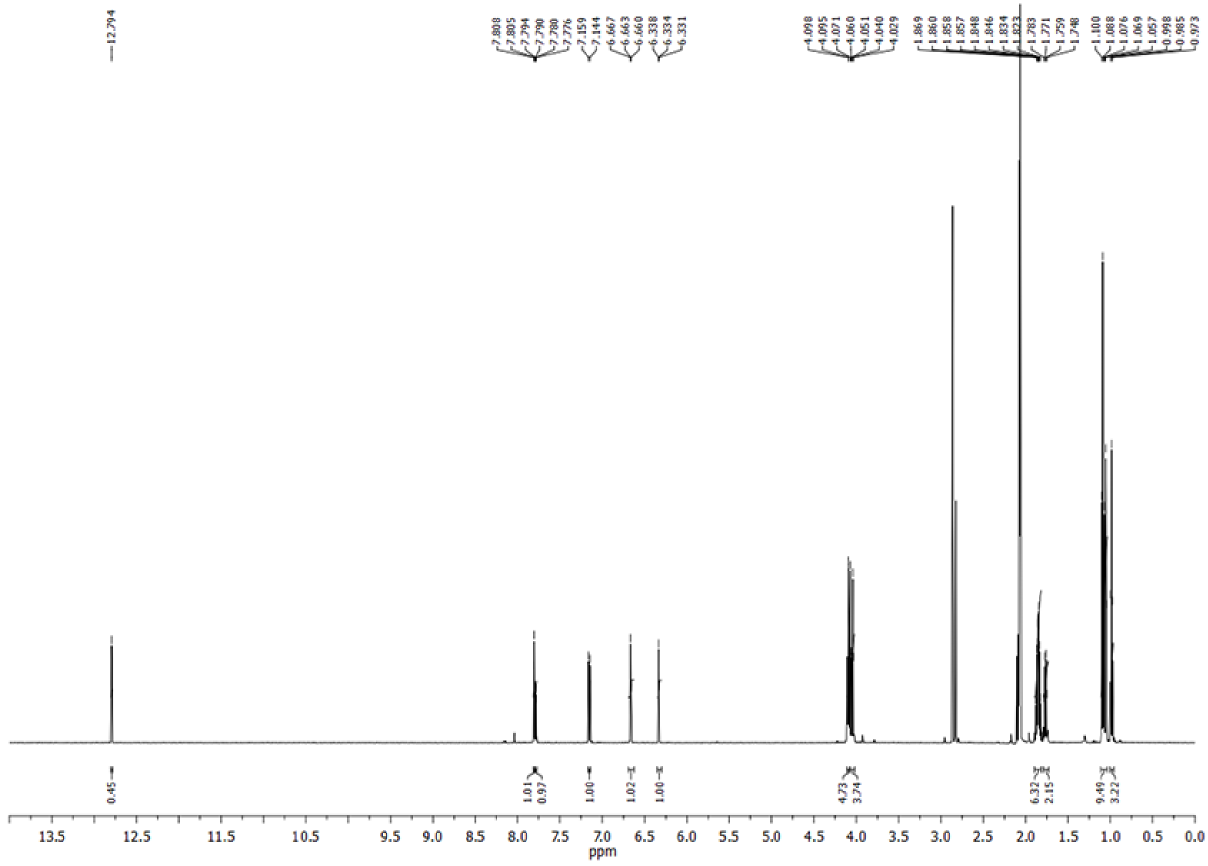

Figure S2. The ^{13}C NMR spectrum of compound **2**

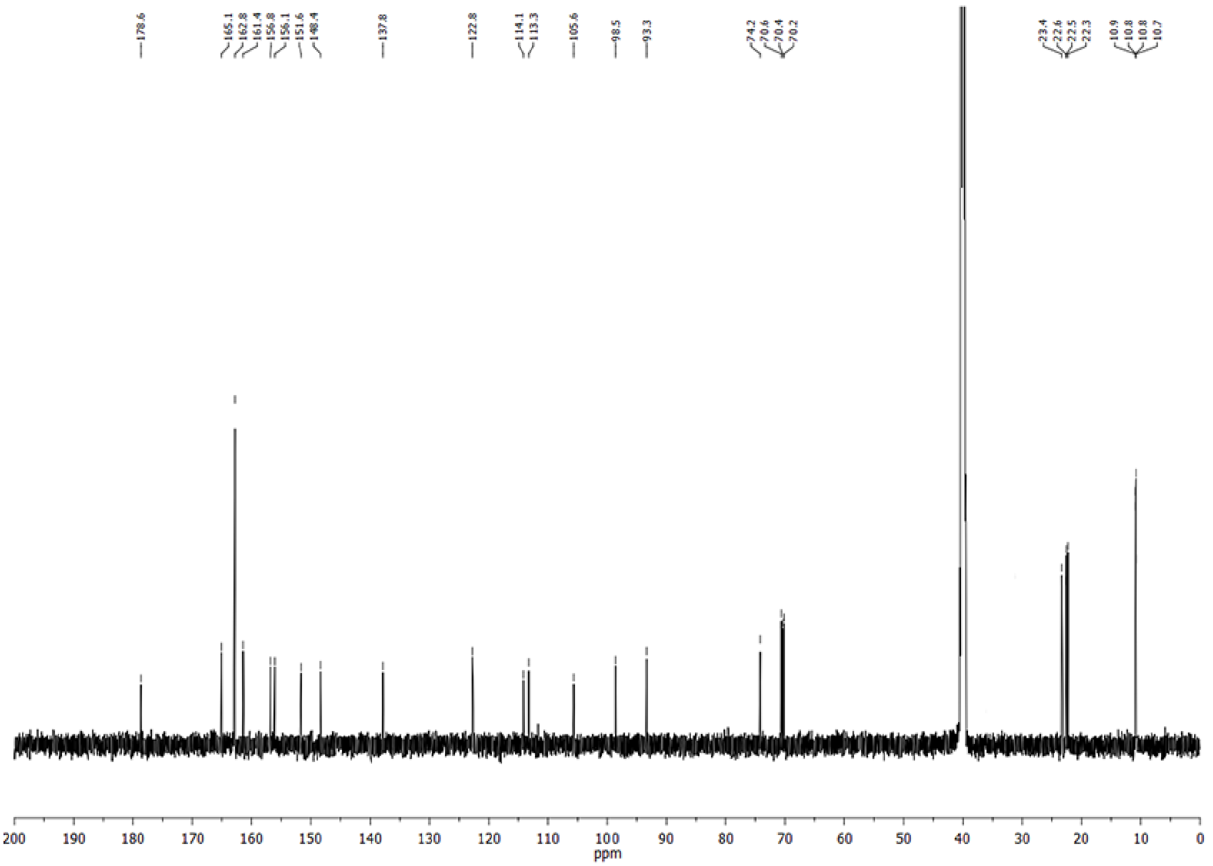

Figure S3. The ^{1}H NMR spectrum of compound **3**

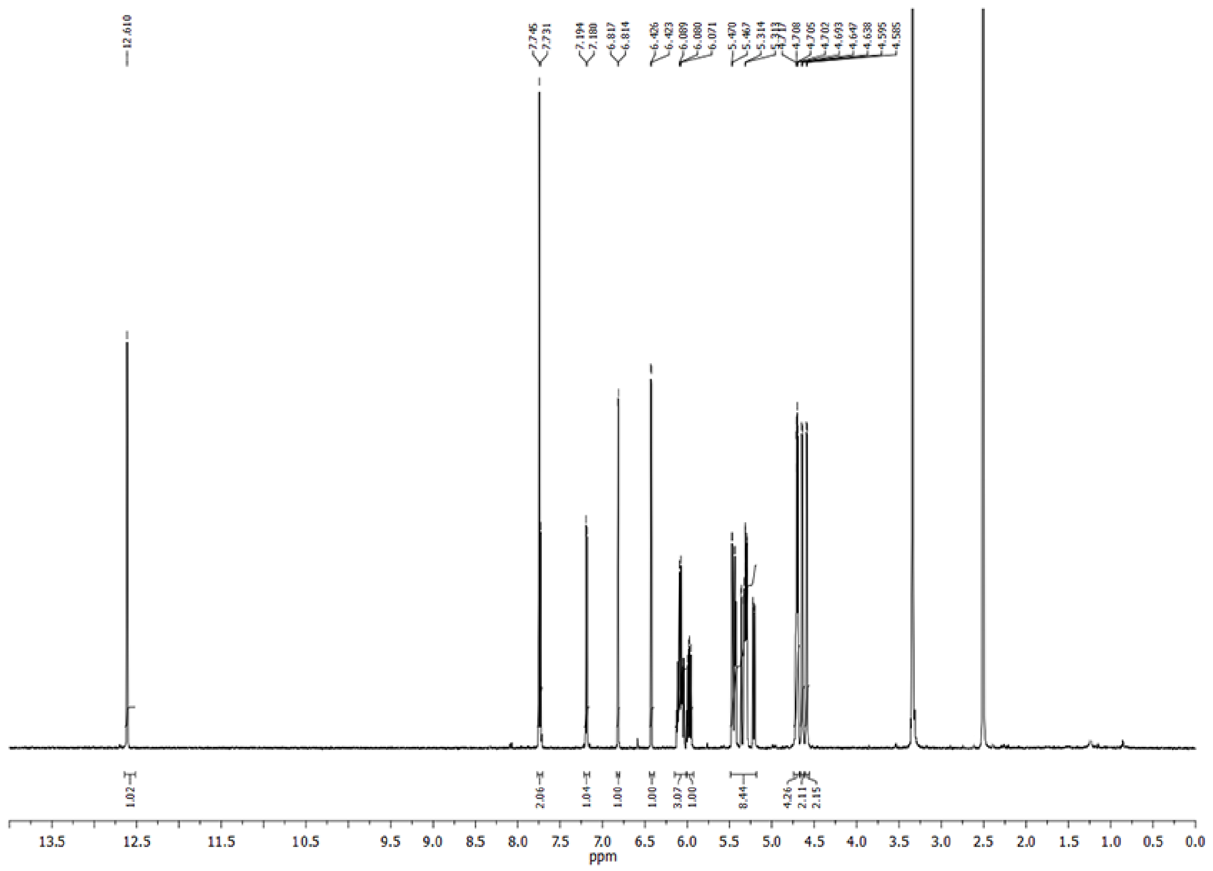

Figure S4. The ^{13}C NMR spectrum of compound **3**

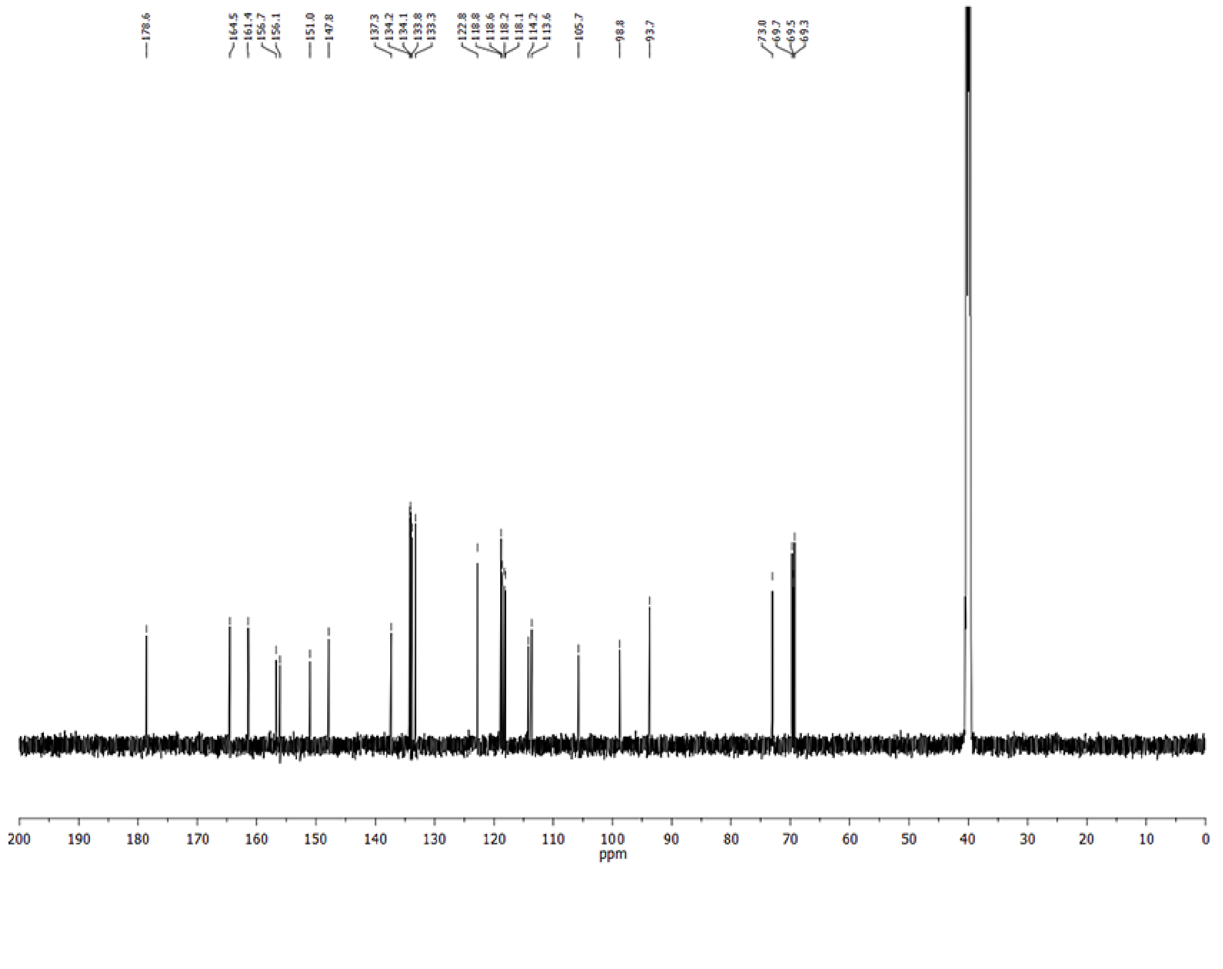

Figure S5. The ^{1}H NMR spectrum of compound **4**

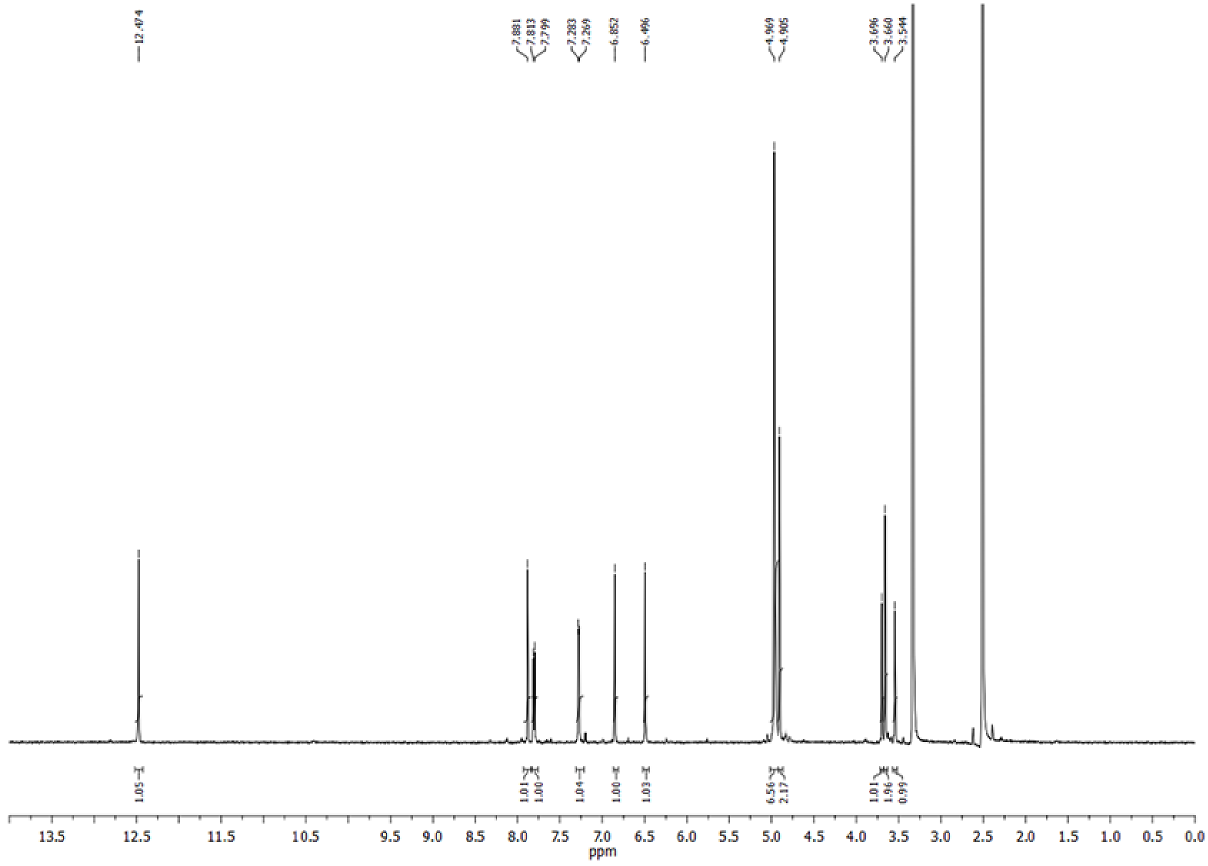

Figure S6. The ^{13}C NMR spectrum of compound **4**

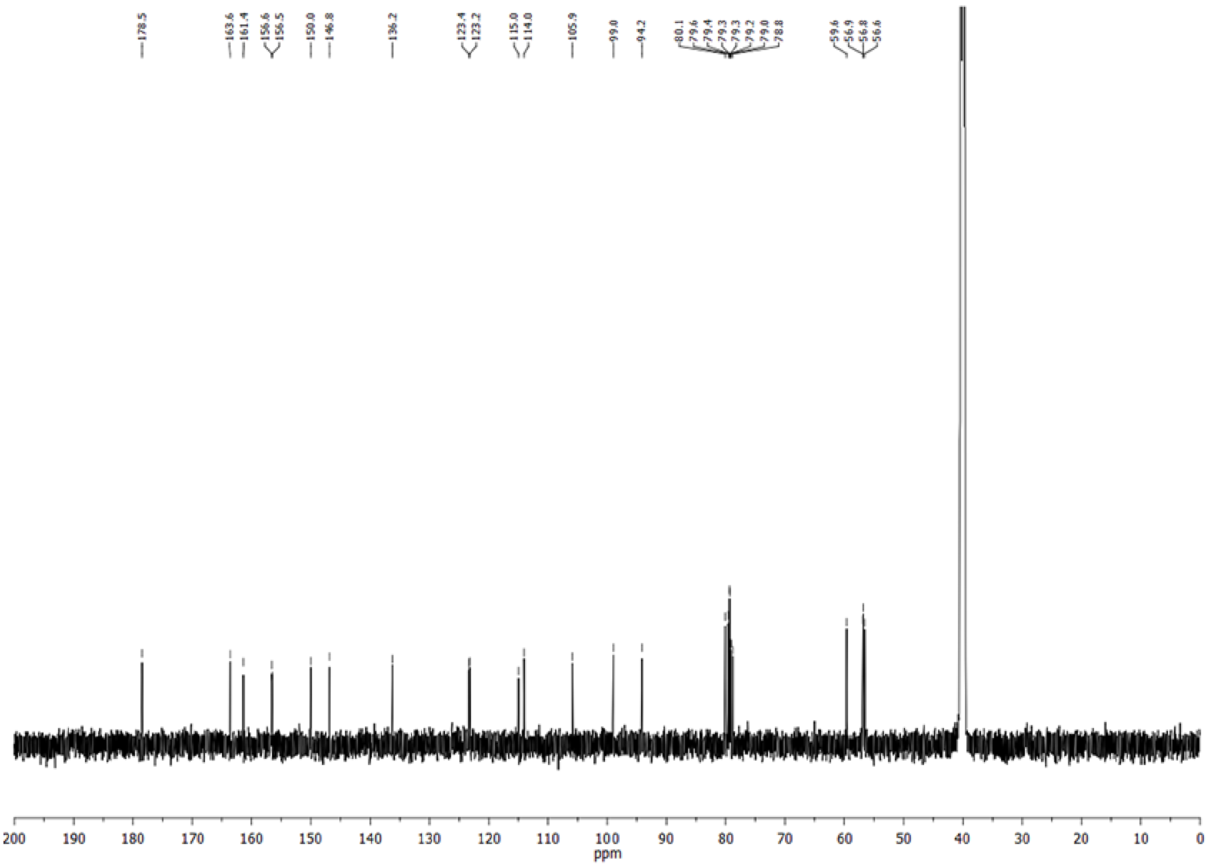

Figure S7. The ^{1}H NMR spectrum of compound **5**

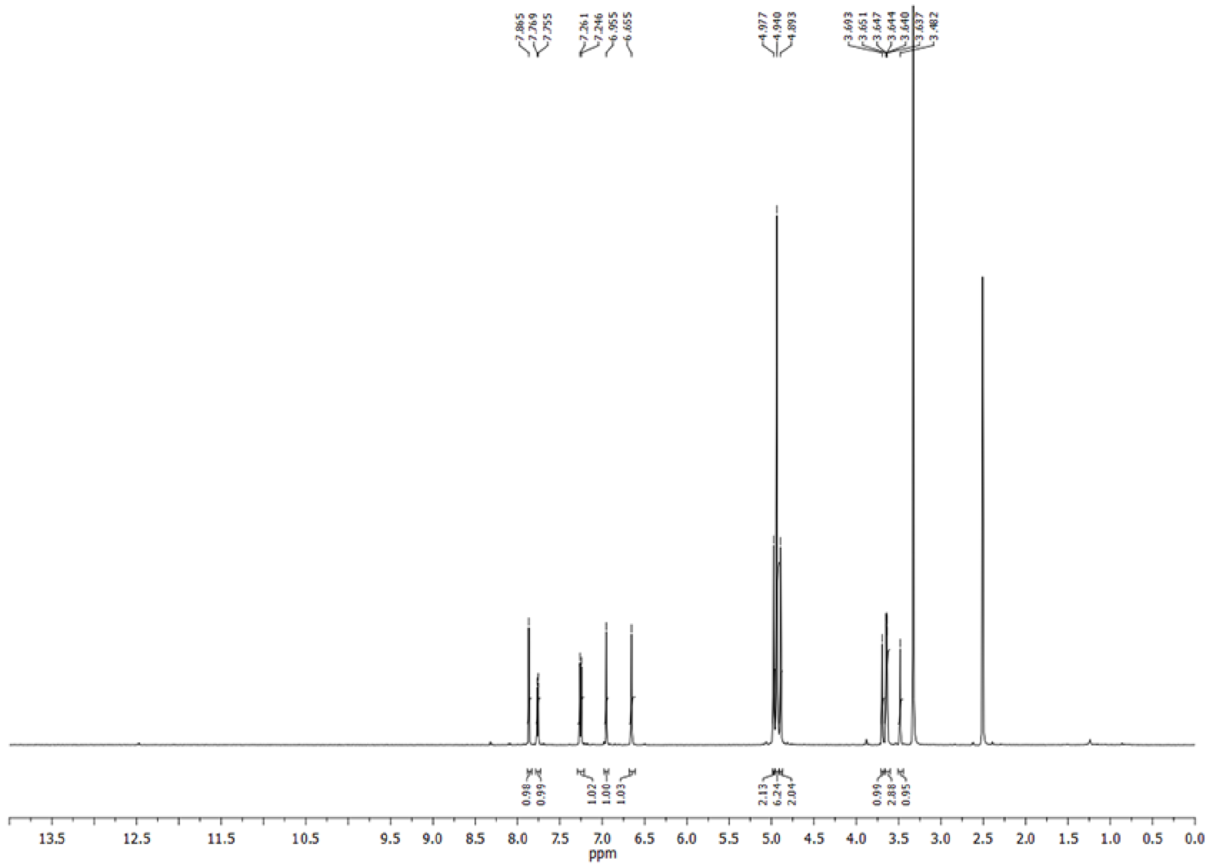

Figure S8. The ^{13}C NMR spectrum of compound **5**

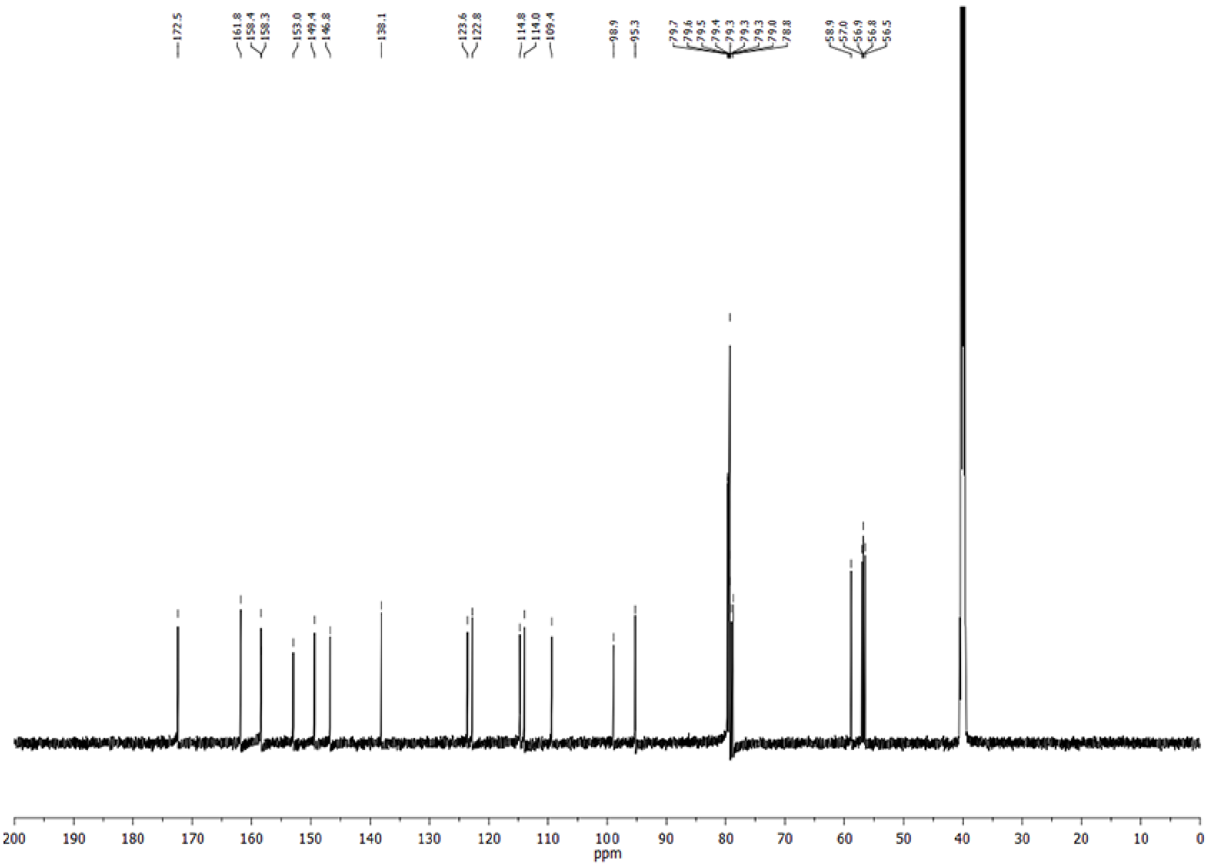

Figure S9 Cytometric analysis of the cell cycle of quercetin and its derivatives

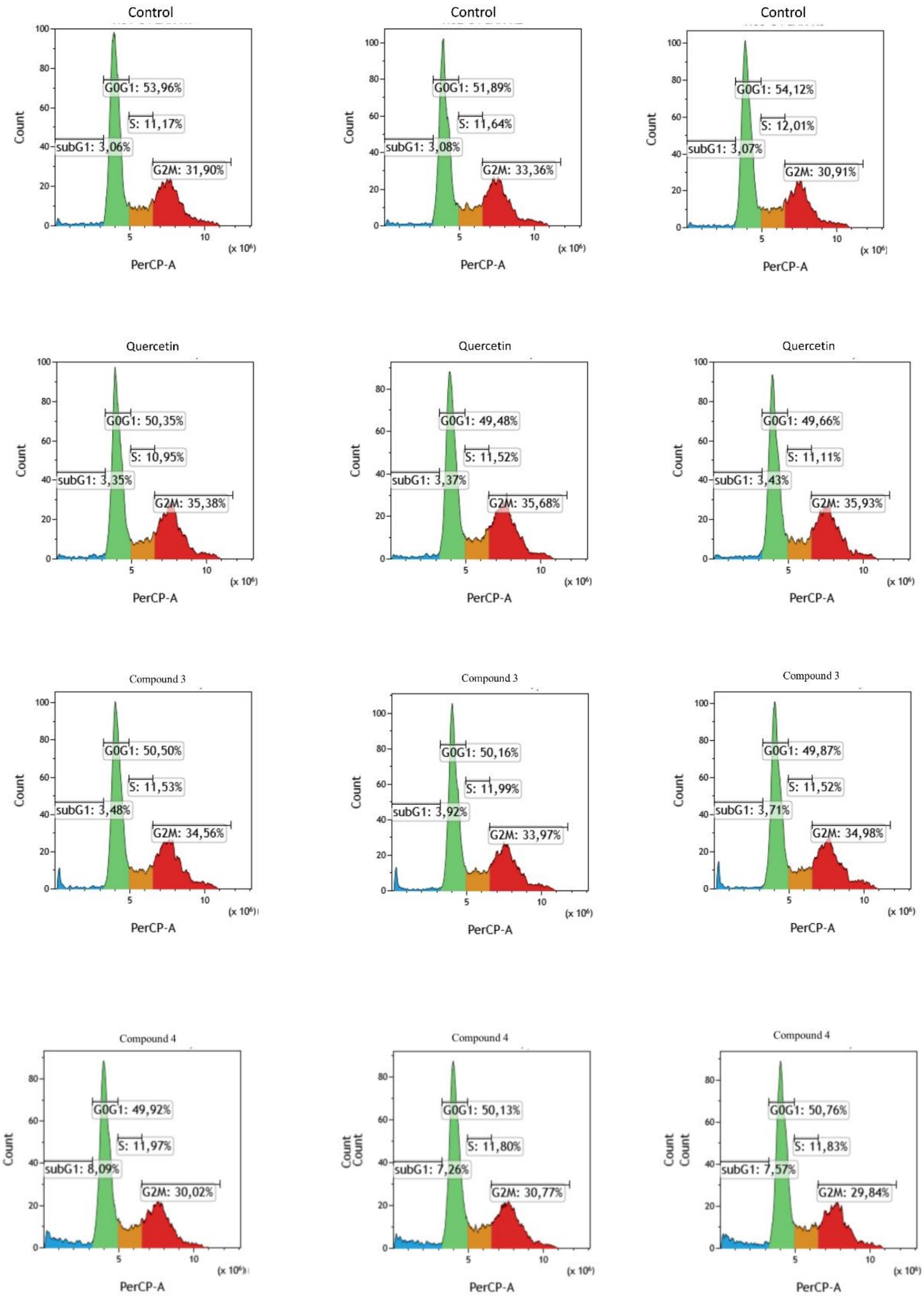

Supplement: Supplementary file 1 — Supplementary Material 1 [file 41598_2026_50954_MOESM1_ESM.pdf]
